# Biographical Sketches of Living Physicians

**Published:** 1865-03

**Authors:** 


### EDITORIAL DEPARTMENT.

### "BIOGRAPHICAL SKETCHES OF LIVING PHYSICIANS."

Publishing biographical sketches of "eminent living physicians" is a new and dangerous method of distinction; and though the victim know nothing of the qualities which are to be awarded him, and is entirely innocent of participation in the exhibition, in no way furnishing "aid and comfort" to his eulogist, nevertheless these panegyrics are offensive to good taste, disgusting to the refined sense of "eminent men," who need no fulsome praise, and are liable to inaugurate in the profession a policy of misrepresentation for most unjustifiable purposes. Biographies of eminent men will not be published to any great extent without the knowledge and consent of the party interested; that is, kindly notices of the life and professional character will not appear without the eulogist or historian being first satisfied that it will be agreeable. Libelous biographical notices, false histories and distorted sketches may possibly appear; indeed there is reason to believe that none of these papers are *true historical records*, but are either overdrawn panegyrics, or untruthful historical records varied according to the individual partiality or prejudice of the author. Some notices of American physicians who are eminently worthy men, have recently appeared in the medical periodicals, and we suppose that in every instance the physician whose biographical history has been thus paraded, has uttered the prayer, if he is a praying man, "O! God, save me from my friends," if not religiously inclined, he may have used some very bad language. The motive for writing these sketehes is plain; every article shows on its face what it is for. The grounds of objection to such papers, are also sufficiently obvious; professional distinction should be gained and heralded in a more legitimate way.

If there are living physicians, who have made important discoveries in science, or who have been eminently connected with great advancements in knowledge of any kind, or have been leaders in the medical revolutions which have within the last few years redeemed, in some degree, our profession from empiricism, we shall be gratified to see these advancements, discoveries and improvements truthfully recorded, and the honor which rightfully belongs to superior merit, properly awarded to those who deserve it. We would like to see this acknowledgment made while yet these men are living, and not altogether reserved for their memories when dead; but we protest against these offerings of partial biographers as indiscreet, untruthful and unprofessional.

Let our eminent men be "known by their works;" if their works are unknown their eminence is conventional, fictitious and undeserved. But we do not question the merit of *eminent living physicians*, we are only opposing the policy of publishing their biographical sketches.

